# *In vitro* and *ex vivo* evaluation of the anti-*Giardia duodenalis* activity of the supernatant of Slab51 (SivoMixx)

**DOI:** 10.1371/journal.pone.0213385

**Published:** 2019-03-07

**Authors:** Stefania Perrucci, Gianluca Fichi, Enrica Ricci, Livio Galosi, Marco Lalle, Giacomo Rossi

**Affiliations:** 1 Department of Veterinary Sciences, University of Pisa, Pisa, Italy; 2 Istituto Zooprofilattico Sperimentale delle Regioni Lazio e Toscana, Pisa, Italy; 3 School of Biosciences and Veterinary Medicine, University of Camerino, Matelica (MC), Italy; 4 Department of Infectious Diseases, Unit of Foodborne and Neglected Parasitic Diseases, European Union Reference Laboratory for Parasites, Istituto Superiore di Sanità, Roma, Italy; National Research Council, ITALY

## Abstract

The effects on *Giardia duodenalis* of Slab51 probiotic supernatants were evaluated *in vitro* and *ex vivo*. *In vitro*, Slab51 (10^1^ UFC) was cultured and the obtained supernatant was filtered, adjusted at pH 7, and added (100μl/ml) as such (Slab51 FS) or after heat-treatment, to *G*. *duodenalis* cultures to evaluate its effects on *G*. *duodenalis* trophozoites growth and adherence. For comparison, negative and metronidazole (20μg/ml) treated controls were used. The morphological and ultrastructural alterations of *G*. *duodenals* trophozoites following treatment with Slab51 FS supernatant were investigated by transmission electron microscopy. *Ex vivo*, mice duodenal portions were cultivated in standard conditions with 5x10^5^ *G*. *duodenalis* trophozoites/ml, while to further five duodenal portions similarly cultured and infected, Slab51 FS 200μl was added. After 12 and 18h, samples were fixed in 10% buffered formalin and histologically processed to score *Giardia* infection and cell damage. Cell proliferation/apoptosis was scored by Ki67, TUNEL and Caspase–3 tests. All experiments were conducted in triplicate throughout the study. All data were statistically evaluated (P< 0.05). Results showed that Slab51 FS significantly reduced *Giardia* growth and adherence respect to negative controls, but its efficacy was overall lower than that of metronidazole. Moreover, the effects of Slab51 FS were significantly lowered by heat-treatment and this reduction was statistically higher at 90°C than at 56°C, indicating a heat-sensitive nature of active Slab51 FS compounds. At the ultrastructural level, Slab51 FS treated *Giardia* trophozoites were swelling, increased in size and showed alterations of their cellular membrane and vacuole patterns, loss of the nuclear envelope and nuclear architecture. In *ex vivo* trials, viable *G*. *duodenalis* trophozoites and enterocyte TUNEL+ and Caspase-3 expression were significantly reduced in intestinal sections added with Slab51 FS, while enterocyte Ki67 expression was significantly increased, confirming the anti-*G*. *duodenalis* activity of Slab51 FS observed *in vitro*. In conclusion, results from this study showed that the fresh culture supernatant of the commercial probiotic Slab51 has anti-*G*. *duodenalis* properties both *in vitro* and *ex vivo* in a mouse model.

## Introduction

Flagellated protozoans of the genus *Giardia* are found in the digestive tract of vertebrate hosts worldwide in which they are the cause of giardiasis [1]. *Giardia duodenalis* (syn. *Giardia intestinalis*, *Giardia lamblia*) is the only species found in humans and in many other wild and domestic mammals worldwide [1, 2]. Based on genetic analysis, *G*. *duodenalis* is considered a species complex, which includes at least eight distinct genetic groups or assemblages, from A to H [1]. Assemblages A and B are usually isolated from humans but can also infect other animals, being considered zoonotic [1, 3].

The localization site of *G*. *duodenalis* is the small intestine, mainly duodenum and jejunum, and it may be responsible for asymptomatic, acute and chronic clinical forms [4, 5]. Diarrhea, malabsorption and weight loss, as well as numerous post-infectious pathologies and extra-intestinal complications are the main clinical signs of symptomatic infections [4–6]. The life cycle of *G*. *duodenalis* is direct and involves two stages, the trophozoite and the cyst. Mammal hosts may acquire *G*. *duodenalis* infections via ingestion of infectious cysts in contaminated food or water sources, or directly via the fecal-oral route [1, 2].

Giardiasis is one of the most common intestinal protozoal infections reported in humans, pet and farm animals [7, 8]. Moreover, human giardiasis has been included in the World Health Organization's (WHO) Neglected Diseases Initiative since 2006, estimating that 280 million people are infected each year [9, 10]. The control of giardiasis is dependent on chemotherapy, and treatments are based mainly on the use of nitroimidazoles, such as metronidazole and tinidazole, and benzimidazoles, mainly fenbendazole and albendazole; furazolidone, acridine, quinacrine, nitazoxanide and paromomycin are also used in some situations [5; 11–15]. However, most of the therapeutically used anti-*Giardia* drugs, including metronidazole, may cause severe side effects and are not well tolerated by many human and animal patients or cannot be used in farm animals [11, 16]. Moreover, the use of these drugs is often associated with clinical failure and drug resistance [16–18]. Hence, identifying new anti-*Giardia* agents is an important consideration for the control of giardiasis in human and veterinary medicine [16, 19].

Some data from recent *in vitro* and *in vivo* studies, largely from mice and humans, have shown that probiotic treatment may possibly ameliorate *G*. *duodenalis* symptoms or reduce infection with *G*. *duodenalis* [6, 20]. These compounds have attracted the attention as potential substitutes for, or as combined therapy to currently used anti-*Giardia* drugs due to their powerful activity, stability and low toxicity to humans and other mammal hosts [11, 21].

In the present study, potential negative effects of the supernatant of a commercial probiotic on *G*. *duodenalis* were evaluated *in vitro* and *ex vivo*.

## Materials and methods

### Slab51 (SivoMixx)

Slab51 (sold in Europe today under the trademark SivoMixx, Ormendes SA, Jouxtens-Mézery, CH) is a commercial multi-strain probiotic containing 200 billion lactic acid bacteria per 1.5 grams of product, comprised of the following strains: *Streptococcus thermophilus* DSM 32245, *Bifidobacterium lactis* DSM 32246, *Bifidobacterium lactis* DSM 32247, *Lactobacillus acidophilus* DSM 32241, *Lactobacillus helveticus* DSM 32242, *Lactobacillus paracasei* DSM 32243, *Lactobacillus plantarum* DSM 32244, *Lactobacillus brevis* DSM 27961.

### Parasite and cultures

Trophozoites (5x10^4^) of *G*. *duodenalis* WB strain (genotype A1) were maintained in axenic culture at 37°C in 8 ml of TYI-S-33 medium in screw-cap cell culture vials. Penicillin G (250μg/ml), streptomycin sulfate (250μg/ml), gentamicin sulfate (50μg/ml) and amphotericin B (0.25μg/ml) were added during routine culture [22, 23]. After two days, log-phase cultures were harvested after cooling the culture vials at 4°C for 15 min and centrifugation at 700 × *g* for 10 min. Trophozoites were washed three times, counted by using a Neubauer cell-counter chamber under light microscope (Nikon Eclipse 80i) and used as inoculum to study the *in vitro* effects of fresh and heat-treated Slab51 supernatants on growth and adherence of *G*. *duodenalis* trophozoites, to evaluate the morphological and ultrastructural alterations of *G*. *duodenalis* trophozoites following treatment with fresh Slab51 supernatant and to evaluate possible *ex vivo* differences between mice intestinal portions cultured with *G*. *duodenalis* trophozoites and with *G*. *duodenalis* trophozoites plus 200μl of fresh Slab51 supernatant.

### Effects of Slab51 supernatant *in vitro*

The effects of Slab51 supernatant on growth and adherence of *G*. *duodenalis* trophozoites were evaluated *in vitro* by using previously reported methods [23–25].

The supernatant was obtained by culturing Slab51 (at 10^1^ UFC) in TYI-S-33 medium without antibiotics at 37°C for 24h and the supernatant (Slab51S) obtained from these cultures was collected, centrifuged at 4,000g x 10min [24], filtered by using filters with pore size of 0.22μm Pes and adjusted at pH 7 by using 5N NaOH. The supernatant was used as fresh (Slab51 FS) and after heat-treatment at 56°C (Slab51S 56°C) and at 90°C (Slab51S 90°C) for 30 minutes.

In all assays, 100μl of Slab51 supernatants were added to 900μl of fresh TYI-S-33 medium in 1.5 ml eppendorf vials with 5x10^4^ log-phase trophozoites (FS-treated groups).

Negative controls (NC) were performed in similar experimental conditions without any supernatants, while positive controls (PC) were performed in similar conditions but adding metronidazole at 20μg/ml to *G*. *duodenalis* culture medium.

To verify the growth of Slab51 lactobacilli in TYI-S-33 medium, Slab51 (10^1^ UFC) was cultured in this medium without antibiotics at 37°C. After 24 h, 100 μl of bacterial colonies grown onto TYI-S-33 media were cultured on MRS agar plates at 37°C. After 24 h, 5 colonies for each plate were identified with API 50CHL (Biomerieux, France).

#### Growth inhibition assay

The growth of *G*. *duodenalis* trophozoites was evaluated at 24 and 48h in cultures treated with fresh (Slab51 FS) and heat-treated (Slab51S 56°C and Slab51S 96°C) Slab51 supernatants, and in negative and positive controls. After each different incubation periods, the culture vials were placed at 4°C for 15min, the trophozoites were resuspended and the total number of cells was counted using a Neubauer cell-counter chamber under light microscope in triplicates (Nikon Eclipse 80i).

#### Adhesion inhibition assay

The effects of Slab51 supernatants (Slab51 FS, Slab51S 56°C and Slab51S 96°C) on the adhesion ability of *G*. *duodenalis* trophozoites were evaluated after 24 and 48h and compared to that observed in negative and positive controls. After inverting to mix, from each culture 10 μl of the medium were removed and the number of unattached cells was counted using a Neubauer cell-counter chamber under light microscope in triplicates (Nikon Eclipse 80i). After exposure to 4°C for 15min, the total cell number was calculated as described in the growth assay. Results were expressed as the percentage of attached trophozoites in relation to the total number of *G*. *duodenalis* trophozoites counted in each culture. More specifically, these percentages were obtained by dividing the difference between the number of trophozoites counted in the medium after exposure to 4°C for 15min (total cells) and the number of trophozoites counted in the medium after mixing at 37°C (non-adhering cells) on the total cells [23].

#### Transmission electron microscopy

After the treatment with the Slab51 supernatant for 24h, the morphological and ultrastructural alterations of *G*. *duodenalis* trophozoites were investigated by transmission electronic microscopy (TEM). To this aim, trophozoite samples were fixed in phosphate-buffered 0.1M of 2% glutaraldehyde (pH 7.4), post-fixed in phosphate-buffered 1% OsO4 and, after dehydration, embedded in Epon/Araldite (Polyscience Inc., Warrington, PA, USA). Ultrathin sections (70 nm) were placed on 200-mesh nickel grids supplied with formvar-carbon film (Agar Scientific Ltd, Stansted, UK). Grids were then stained with lead citrate and uranyl acetate and examined with a JEOL 1200-EX transmission electron microscope (JEOL, Peabody, MA, USA).

### Effects of Slab51 *ex vivo*

With the aim to evaluate the anti-*Giardia* activity of Slab51 FS supernatant in controlled conditions but with the minimum alteration of natural conditions, some *ex vivo* trials were conducted on mice gut. Intestinal tracts were taken from healthy mice used as negative control in a study approved by the institutional research ethics board of the Italian Ministry of Health (authorization n°244/2017-PR). More specifically, thirty– 1 cm long duodenal portions were taken from CD-1(ICR)BR mice obtained from Charles River GmbH, Sulzfeld, Germany. Animals were kept according to the Italian regulations of animal experiments with free access to germfree food and sterile water. All mice were considered negative for *Giardia* spp. infection based on the absence of *Giardia* trophozoites, cysts and fecal antigens in three fecal samples collected from each mouse in three non-consecutive days and examined by fresh and Lugol stained fecal smears, flotation test and a commercial rapid immune-chromatographic assay (RIDA QUICK *Cryptosporidium*/*Giardia* Combi, R-Biopharm, Darmstadt, Germany) [26]. Mice duodenal portions were cultivated *in vitro* for 12 to 18h in RPMI 1640 medium containing 10% v/v heat-inactivated fetal bovine serum added with 100 units/ml of an antibiotic-antimycotic solution (Antibiotic Antimycotic Solution, Sigma-Aldrich, Saint Louis, MO, USA). Specimens were then placed in 25ml Falcon’s tubes and incubated at 37°C with 5% CO_2_ until examination. Five intestinal fragments were cultured with 1.8ml of medium containing 5x10^5^ *G*. *duodenalis* WB strain trophozoites/ml plus 200μl of ultrafiltered Slab51 FS, while further five intestinal fragments were cultured with 1.8ml of the same medium containing 5x10^5^ trophozoites/ml plus 200μl of sterile saline solution (negative controls, NC). Samples were stopped at different times (12 and 18h, respectively) and the tissues fixed in 10% buffered formalin for a period of 8h, then washed in sterile saline solution, dehydrated and paraffin embedded.

#### Histological examination

Two-μm paraffin sections were placed on Superfrost Plus slides (Histoline, Milan, Italy). The slides were then dewaxed and stained with hematoxylin & eosin stain (H&E) for microscopic examination, primarily to score the intensity of infection and the morphology of the intestinal mucosa at different time periods both in Slab51 FS-treated and in untreated samples, as reported afterwards. Histological examination included assessment of inflammation by scoring the number of inflammatory cells (mononuclear cells, such as macrophages, lymphocytes, and plasma cells, and neutrophils) at a magnification of ×400. The number of inflammatory cells was evaluated by using a visual analogue scale modified for murine gastrointestinal specimens, and results were reported as the mean for the entire specimen. When considerable variation of intensity of infiltration was evident in the same specimen, the mean for several areas was determined and the specimen was scored accordingly. Neutrophils and mononuclear cells were classified as absent (score of 0) when at a magnification of ×400, there were no or fewer than 5 cells per high-power field (HPF), mild (score of 1) for 5 to 19 cells per HPF, moderate (score of 2) for 20 to 49 cells per HPF, marked (score of 3) for 50 to 99 cells per HPF, and severe (score of 4) for 100 to 200 cells or more per HPF.

Histological criteria for normal intestinal characteristics included detection of no or only a few mononuclear cells per HPF and no or only a few scattered neutrophils in sub-epithelial areas and/or in peri-glandular area of duodenal mucosa, without tissue changes, i.e. no interstitial thickening, Peyer’s patches/gut associated lymphoid tissue [GALT] enlargement or epithelial-associated lymphocytes increase).

#### Immuno-histochemical tests

Paraffin sections were used for immuno-histochemical tests. Rehydrated sections were treated for endogenous peroxidases neutralization with 3% hydrogen peroxide for 1h followed by rinsing for 5min in deionized water. Antigen retrieval was achieved by incubating slides in antigen retrieval solution in a steamer (Black & Decker, Towson, MD, USA) for 20min. Nonspecific immunoglobulin binding was blocked by incubation of slides for 10min with a protein-blocking agent (Dako, Carpinteria, CA, USA) before application of the primary antibody. Slides were incubated overnight in a moist-chamber with polyclonal rabbit anti-human Ki67 antibody (Santa Cruz Biotechnology, Inc., Dallas, TX, USA), used as primary antibody at dilutions of 1:50. A goat anti-rabbit byotinilated antibody (Dako), was used as secondary antibody at standard dilution of 1:250 in buffer. A streptavidin–immunoperoxidase staining procedure (Dako, Carpinteria, CA, USA) was used for immunolabeling. The immunoreaction was observed with 3,3'-diaminobenzidine (DAB) or VIP substrate (Vector Laboratories, Inc., Burlingame, CA, USA). Sections were counterstained with Mayer's hematoxylin. Positive immunohistochemical controls included mouse mammary carcinoma sections. Negative immunohistochemical controls were known mouse mammary carcinoma or intestinal sections, treated identically as routine sections with 20min antigen retrieval and 10min protein blocking, except that the overnight incubation with primary antibodies was replaced by an overnight incubation with buffer. Expression of cleaved Caspase-3 in paraffin-embedded tissue sections was investigated using the Anti-active Caspase-3 antibody (Promega Corporation, Madison, WI, USA) directed against a peptide from the p17 fragment of the active (cleaved) human Caspase-3, and after an O/N incubation with this antibody, sections were treated routinely as described above. Sections from mouse mesenteric lymph nodes were subsequently selected as the positive control for further tests. The primary antibody was replaced by phosphate buffered saline solution (PBS) as a negative control. In small intestinal sections, pro-apoptotic effect induced by Giardia in crypts, and mucosal lining epithelial cells were highlighted through a TUNEL colorimetric staining (DeadEnd, Promega Corporation, Madison, WI, USA) according to the manufacturer’s instructions. To score the intensity of *G*. *duodenalis* trophozoites, and Ki67 positive cells at different times in treated and untreated samples, ten random fields of the sample were examined under a dry-X40 objective. The total number of protozoans and Ki67 stained epithelial cells was recorded. The mean value obtained per histological section per time was considered. To enumerate the TUNEL positive nuclei and cleaved Caspase-3 expression, tissues were graded in five categories by two independent blinded observers according to the number of detected apoptotic cells as follows: 0: without any apoptotic signal; 1: low level of apoptotic signal (<5%); 2: moderate level of apoptotic signal (5–10%); 3: high level of apoptotic signal (10–20%); 4: very high level of apoptotic signal (>20%). For the evaluation of these parameters indicating the apoptotic rate, 10 random fields of the sample were examined under a dry-x40 objective. The number of positive enterocytes was normalized to the number of enterocytes per field and expressed as a percentage of these values. Similarly, Ki-67 slides were visually scanned and scored: 0, negative (<5% positive cells); 1, sporadic (5%-20% positive cells); 2, moderate (20–50%); 3, diffuse (50–75%), and 4, strongly diffuse (>75% cells). For all parameters, cells on the margins of the tissue sections were not considered for evaluation to avoid possible artifactual staining.

### Statistical analysis

All *in vitro* experiments were repeated in triplicate in two independent assays. Values were expressed as mean ± sd and compared by repeated measures analysis of variance, followed by Bonferroni’s multiple comparison [23].

All *ex vivo* experiments were repeated in triplicate. Descriptive and comparative statistical analyses of *ex vivo* tests were performed and results (mitotic and apoptotic cell numbers and number of *G*. *duodenalis* trophozoites in fresh supernatant-treated or untreated tri-dimensional culture cells) were described and tested for normal distribution with the Kolmogorov–Smirnov test and normal probability plots. As they were not normally distributed, the non-parametric Wilcoxon Signed-Rank test was used to compare median values for these variables between the treated and untreated control biopsies. Correlations between degrees of expressions of these different variants in the two groups of samples were analysed with Spearman rank tests [27].

The level of statistical significance was set at P <0.05 throughout the study.

## Results

### Effects of Slab51 *in vitro*

**Fig 1:**
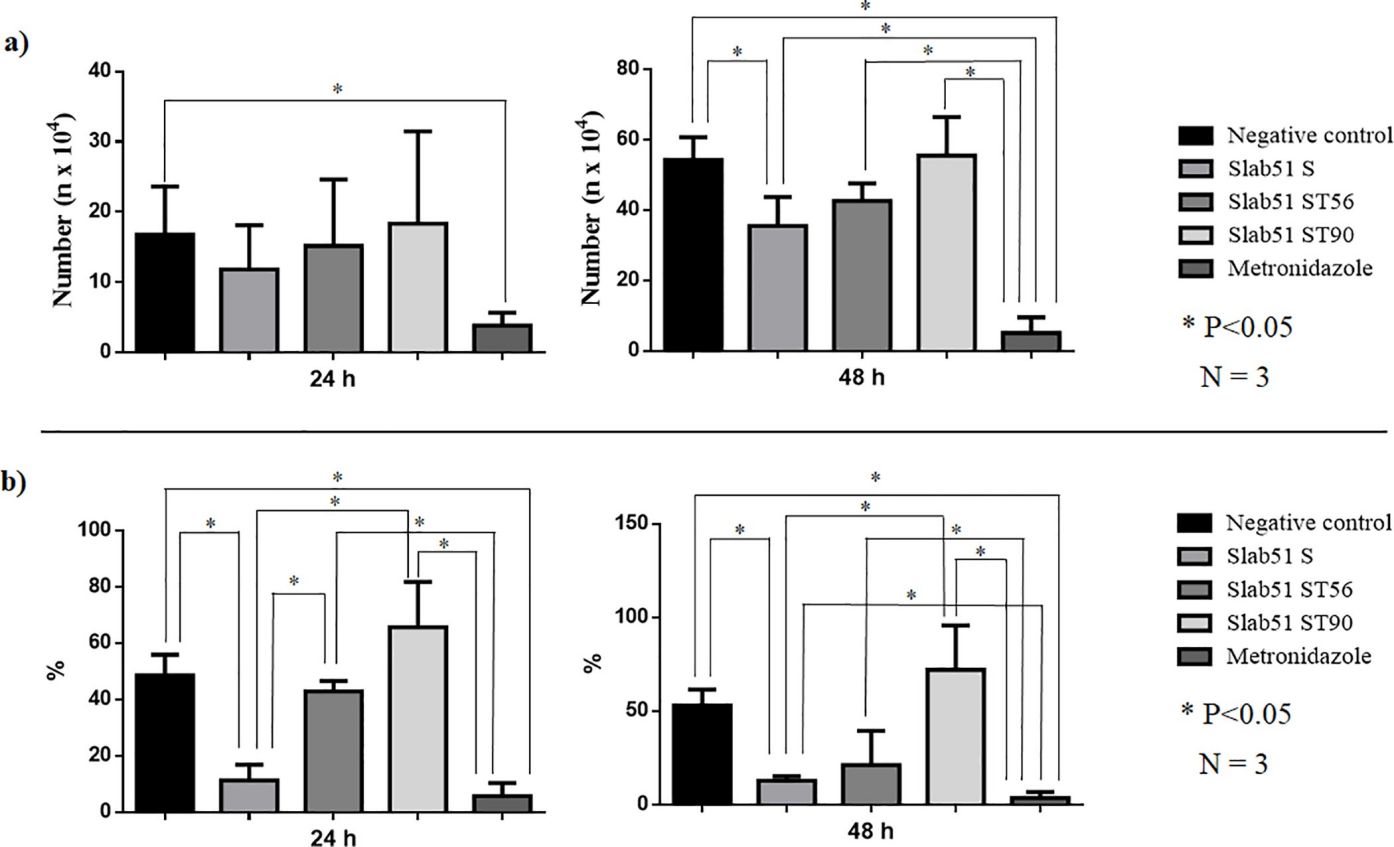
(**a**) Growth inhibition of *Giardia duodenalis* trophozoites by fresh (Slab51 FS) and 56°C (Slab51S 56°C) and 90°C (Slab51S 96°C) heat-treated Slab51 supernatants. The number (n x10^4^) of *G*. *duodenalis* trophozoites are expressed as average and standard deviation of trophozoites counted in three replicates after 24 and 48 h observation periods.; (**b**) Adhesion inhibition of *G*. *duodenalis* trophozoites by fresh (Slab51 FS) and 56°C (Slab51S 56°C) and 90°C (Slab51S 96°C) heat-treated Slab51 supernatants. Attached trophozoites have been expressed as the mean percentage of attached *G*. *duodenalis* trophozoites in relation to the total number of *G*. *duodenalis* trophozoites counted after 24 and 48 hours in each culture and in three replicates.

Slab51FS was able to inhibit the growth and the adhesion ability of *G*. *duodenalis* trophozoites respect to untreated controls, but its effects were generally lower than that of metronidazole, the reference drug. In fact, in the growth inhibition assay the number of trophozoites (35.48±8.16x10^4^) found after 48h in cultures treated with fresh Slab51 supernatant was significantly (P <0.05) reduced respect to that counted in untreated cultures (54.15±6.58x10^4^). However, this reduction was significantly lower (P <0.05) respect to that observed in cultures treated with metronidazole where the number of trophozoites was extremely low (5.05±4.47x10^4^) (Table 1; Fig 1). In the adhesion assays, after 24h the number of adherent *G*. *duodenalis* trophozoites (9.51± 7.08%) observed in Slab51 FS-treated cultures was significantly reduced (P <0.05) respect to that observed in untreated cultures (48.58± 7.31%) but similar to that counted in culture treated with metronidazole (5.64±4.75). However, after 48h the number of adherent *G*. *duodenalis* trophozoites (12.85±2.26%) counted in Slab51 FS-treated cultures was still significantly lower (P<0.05) respect to that observed in untreated cultures (52.91±8.64%), but significantly higher (P<0.05) than in culture treated with metronidazole (3.43±3.44%) (Table 2; Fig 1). On the contrary, the cultures treated with metronidazole showed a significant reduction of the growth and adhesion of *G*. *duodenalis* trophozoites in comparison with untreated cultures, both at 24h and at 48h (Tables 1 and 2; Fig 1).

**Table 1:** Growth inhibition of *Giardia duodenalis* trophozoites by fresh (Slab51 FS) and 56°C (Slab51S 56°C) and 90°C (Slab51S 96°C) heat-treated Slab51 supernatants. The number (n x10^4^) of *Giardia duodenalis* trophozoites are expressed as average and standard deviation of trophozoites counted in three replicates after 24 and 48 h observation periods.

Growth assay				
	24 h		48h	
	Mean	SD	Mean	SD
**Negative control**	16.74^b^	6.88	54.15^c^	6.58
**Slab51 FS**	11.82^a^^b^	6.26	35.48^b^	8.16
**Slab51 S 56**°C	15.14^a^^b^	9.46	42.54^b^^c^	5.02
**Slab51 S 90°C**	18.32^a^^b^	13.16	55.42^b^^c^	10.96
**Metronidazole**	3.82^a^	1.82	5.05^a^	4.47

a,b,c: P < 0.05

**Table 2:** Adhesion inhibition of *Giardia duodenalis* trophozoites by fresh (Slab51 FS) and 56°C (Slab51S 56°C) and 90°C (Slab51S 96°C) heat-treated Slab51 supernatants. Attached trophozoites have been expressed as the mean percentage of attached *G*. *duodenalis* trophozoites in relation to the total number of *G*. *duodenalis* trophozoites counted after 24 and 48 hours in each culture and in three replicates.

Adhesion assay				
	24 h		48h	
	Mean	SD	Mean	SD
**Negative control**	48.58^b^	7.31	52.91^c^	8.64
**Slab51S**	9.51^a^	7.08	12.85^b^	2.26
**Slab51S 56°C**	42.84^b^	3.70	21.15^b^^c^	18.33
**Slab51S 90°C**	65.65^b^	16.10	72.06^c^	23.79
**Metronidazole**	5.64^a^	4.75	3.43^a^	3.44

a,b,c: P < 0.05

The heat-treatment reduced the negative effects on *G*. *duodenalis* growth and adhesion ability of fresh Slab51 supernatant. In fact, while after 48 h the inhibiting activity of 56°C heat-treated Slab51 supernatant (42.54± 5.02%) was significantly different (P<0.05) both from treated (5.05±4.47x10^4^) and untreated controls (54.15±6.58 x10^4^), as well as from Slab51 FS-treated cultures (35.48±8.16x10^4^). After the same time-period, results observed for 90°C heat-treated Slab51 supernatant cultures (55.42±10.96 x10^4^) were comparable to that of untreated controls (Table 1, Fig 1). Moreover, in the adhesion assay no statistical difference with untreated cultures (52.91±8.64%) was observed both for 56°C (21.15±18.33%) and 90°C heat-treated Slab51 supernatant cultures (72.06±10.96%) (Table 2).

All the colonies from Slab51 cultures in TYI-S-33 medium with and without antibiotics and cultured in MRS agar plates were identified as *L*. *plantarum*.

**Fig 2:**
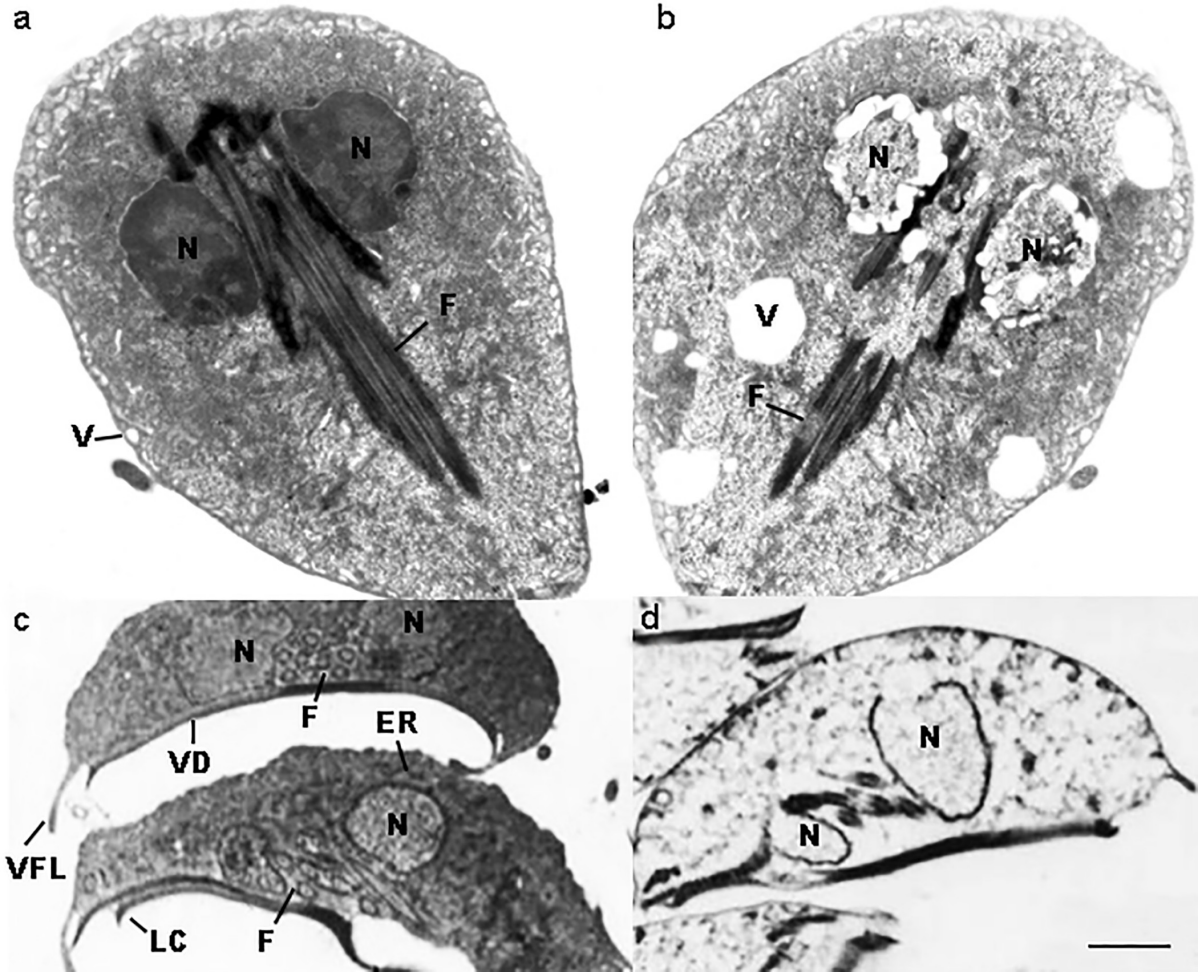
Ultrastructure of *Giardia duodenalis* by TEM. Untreated *G*. *duodenalis* trophozoites showing normal structure and morphology (**a**). Trophozoite coronal section (**c**). A coronal view of a trophozoite demonstrates the nuclei (N), endoplasmic reticulum (ER), flagella (F), vacuoles (V), ventral disk (VD), lateral crest (LC) and ventrolateral flange (VLF). The same sections (**b, d**) of treated parasites show swelling trophozoites, with an increased size, and evident alterations of their cellular membrane and with a vacuolar degenerative pattern (x6,700). Note in the coronal section the severely damaged nuclei, nuclear membrane rupture, loss of the chromatin, flanges and ventral disk rupture (x6,700).

At the ultrastructural level, untreated trophozoites showed normal structure and morphology (Fig 1A and 1C), while treated parasites were swelling and increased in size (Fig 2B and 2D). Moreover, trophozoites showed alterations of their cellular membrane and vacuole patterns. Inside the cells, an ostensibly low electron density and granules grouped in clusters were evidenced. In the nucleus, the loss of the nuclear envelope and nuclear architecture and the presence of structures resembling holes or lacunas were clearly visible (Fig 2B and 2D).

### Effects of Slab51 *ex vivo*

**Fig 3:**
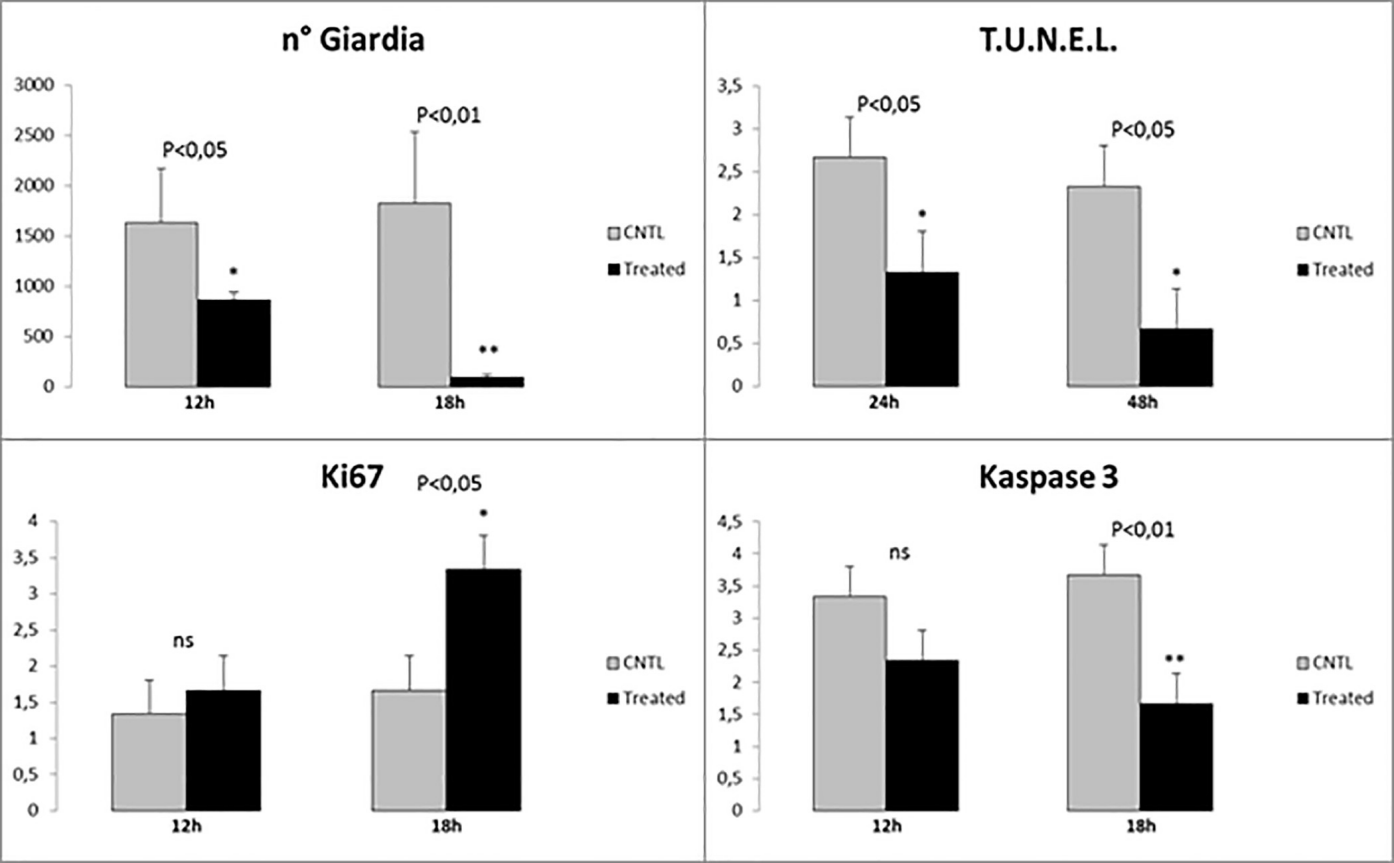
Statistical evaluation of different parameters in *ex-vivo* duodenal tissue cultures, before and after treatment. (**a**) Significant reduction in the number of viable *Giardia* cells counted in biopsies treated with Slab51 ultrafiltered supernatant at 12h and 18h. (**b**) Statistical comparison of TUNEL positive nuclei before and after the same treatment at 24h and 48h. (**c**) Statistical comparison of level of cellular viability and replication by Ki67 nuclear assessment at 12h and 18h. (**d**) Statistical confrontation of Caspase3 positive cells before and after the same treatment at 12h and 18h. Caspase3 expression in association with the level of the TUNEL expression, as showed in Fig 2B, indicate the entire fraction of apoptotic cell because these two apoptotic markers are expressed in subsequent time.

**Fig 4:**
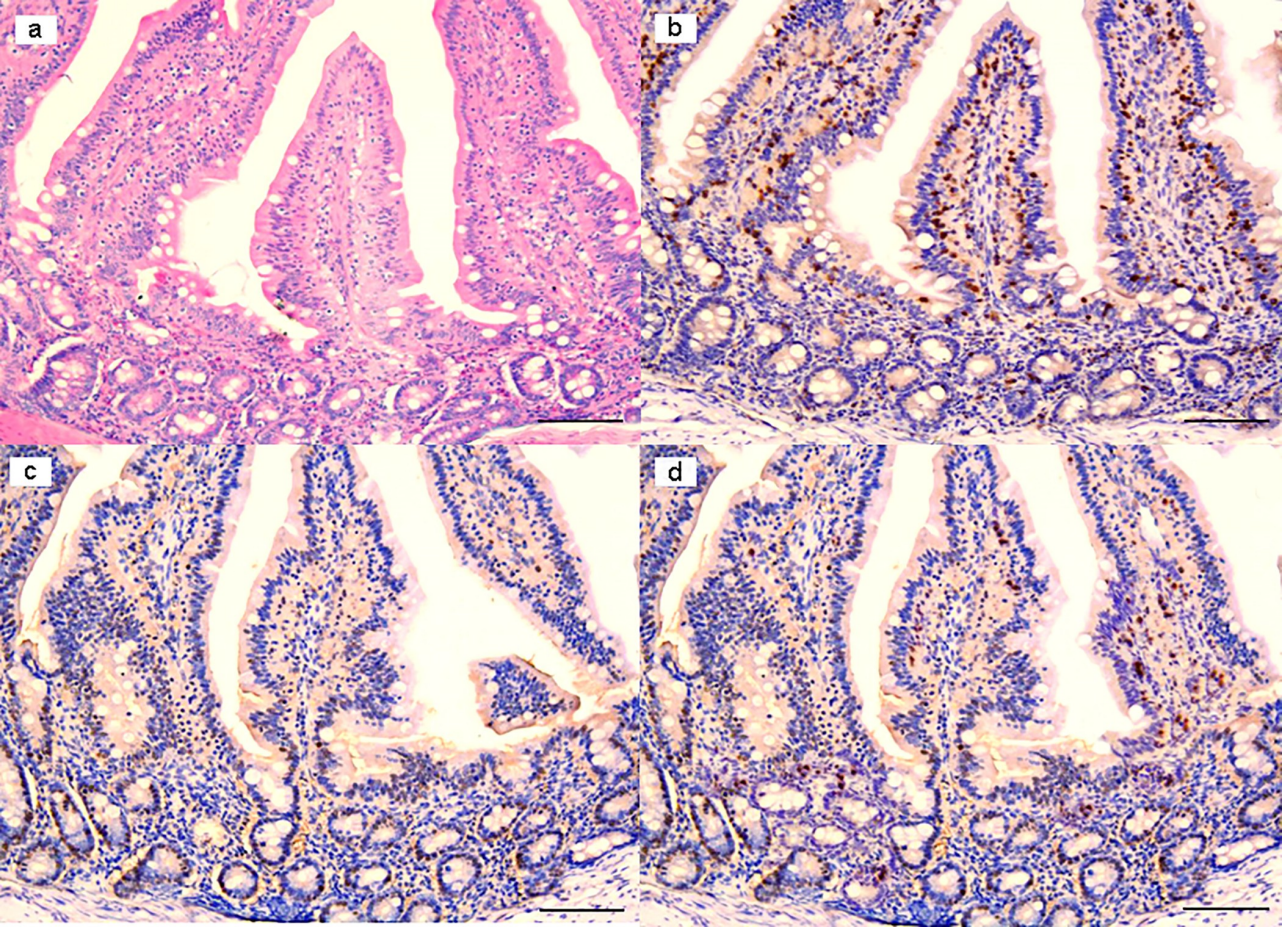
*Ex-vivo* intestinal tissue, from mice treated with Slab51 ultrafiltered fresh supernatant. At 18h post-infection with *Giardia duodenalis*, biopsies showed a preserved morphology and viability as demonstrated by H&E stain (**a**) and Ki67 enterocytes expression (**b**). In these samples, a low number of TUNEL+ enterocytes is observed (**c**). A similar pattern of expression of Caspase-3 indicates a low apoptotic rate in these samples (**d**). Presence of inflammatory cells with a diffuse and non-polarized pattern of infiltration is also observed in these biopsies (H&E, and IHC with Mayer Haematoxilin nuclear counterstain, scale bar 400 μm).

**Fig 5:**
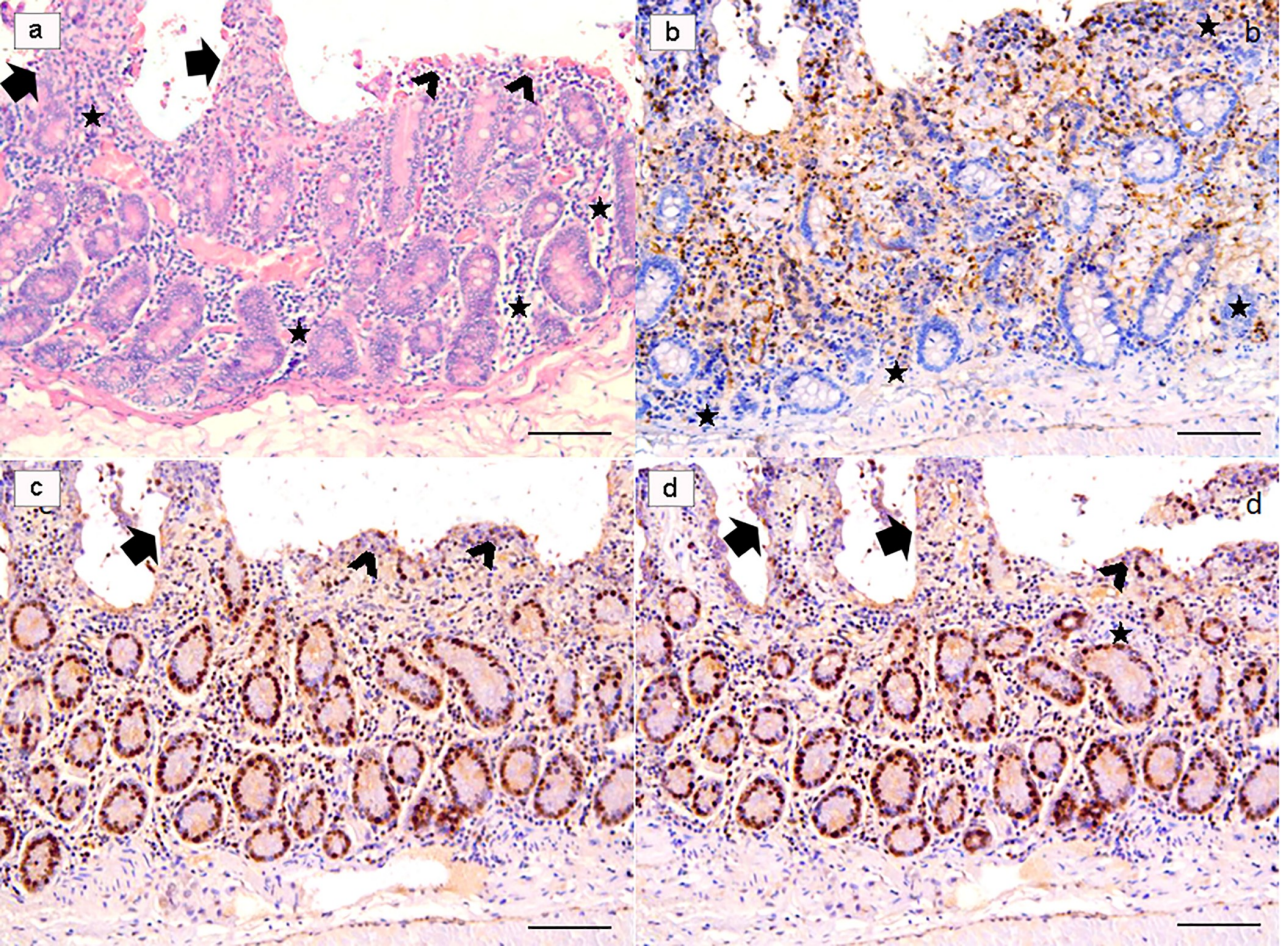
*Ex-vivo* intestinal tissue, from negative control (NC) group. Section stained with H&E revealed a diffuse epithelial loss with inflammatory cells infiltration polarized under the destroyed intestinal epithelium. Note the reinforcement of inflammatory cells around intestinal glands (**a**). Villi are partially damaged (arrows), while in part they are totally flat or in any case strongly tuned (arrowheads) due to the effect of the strong colonization-adhesion of *Giardia duodenalis* trophozoites on the surface of the epithelium, which has become detached in many areas of the mucosa. Note the reinforcement of inflammatory cells around intestinal glands (stars). Ki67 nuclear staining evidenced an apparently higher number of positive cells because many inflammatory cells showed a strongly nuclear positivity (**b**). Note that TUNEL (**c**) and Caspase-3 (**d**) are over-expressed in these explanted intestinal samples. (H&E, and IHC with Mayer Haematoxilin nuclear counterstain, scale bar 400 μm).

**Fig 6:**
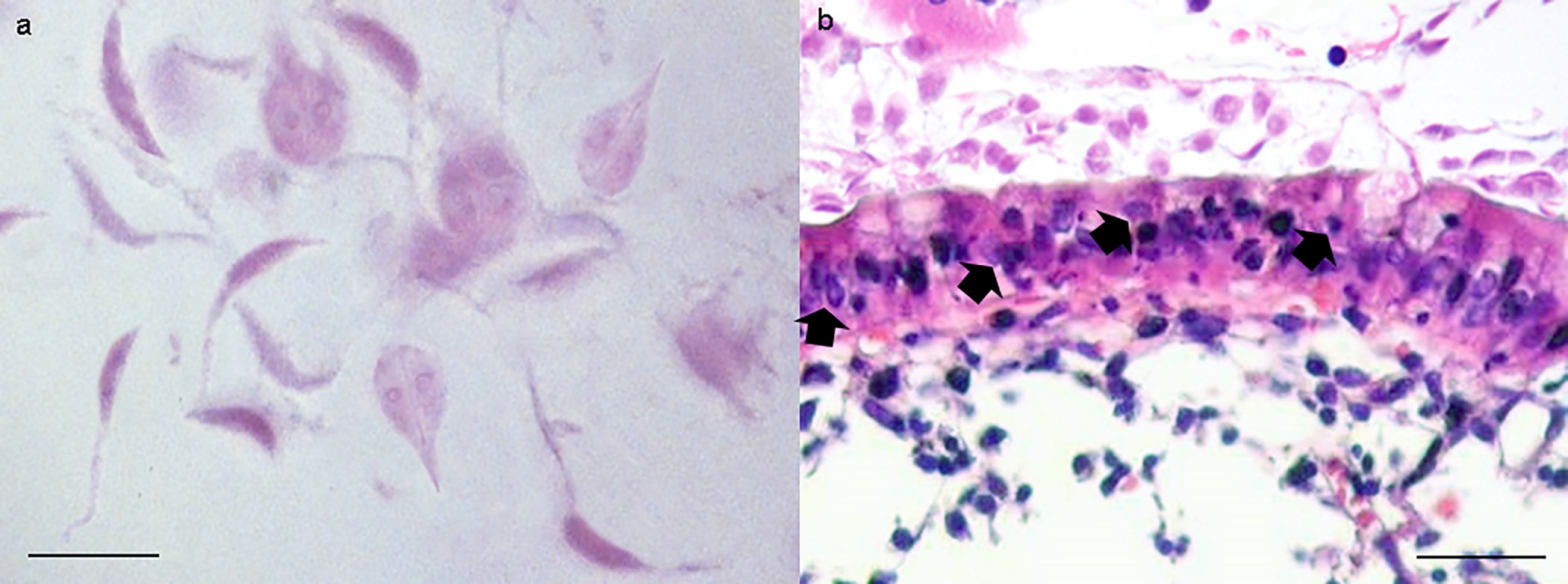
*Giardia duodenalis* in negative control (NC) group. *G*. *duodenalis* trophozoites showed an intact morphology also after 18h (**a**), and the apoptosis rate of enterocytes (arrows), as demonstrated in **b**, increased progressively during the experiment, by the combined effect of the infection and the *ex-vivo* condition. (H&E, scale bar: a, 10 μm–b, 50 μm).

In *ex vivo* trials, significant results were observed in treated samples at 18h post-infection (PI). Indeed, at this time treated with Slab51 ultrafilterd fresh supernatant showed a significant reduction of viable *G*. *duodenalis* trophozoites at the end of the observation period, as evidenced in Fig 3. In these same samples, a progressive and significant decrease in TUNEL+ enterocytes was observed (Fig 4C), while at the same time apoptotic activity peaked in untreated samples (Fig 5C) when compared to Slab51 FS-treated samples. Similar results were obtained in sections stained for Caspase-3 (Figs 4D and 5D). In fact, as shown in Fig 4D, also for Caspase-3 the peak in decrease of expression was observed in *ex vivo* intestinal tissue cultures after 18h of incubation with Slab51 ultrafilterd fresh supernatant. In untreated controls, *G*. *duodenalis* trophozoites showed an intact morphology also after 18h (Fig 6A) while the apoptosis rate of enterocytes in untreated samples increased progressively in these groups throughout the experiment (Fig 6B). As shown in Figs 3B and 4B, Ki67 enterocyte nuclear staining was observed in mice intestinal mucosa still after 18h in both Slab51 FS-treated groups and untreated control groups, suggesting that even after this period of cultivation, the epithelium covering the intestinal mucosa is still able to live and proliferate *ex vivo*. These observations were also supported by the general morphology of bioptic samples. In fact, even if well preserved, untreated samples showed a clear loss of epithelial cells with a different pattern of inflammatory cell distribution throughout the mucosal corion, as evidenced in haematoxylin-eosin stained tissues (Figs 4A and 5A).

## Discussion

*G*. *duodenalis* is a common fecal-oral parasite of the small intestine and one of the most important causes of human and animal diarrheal disease worldwide. Indeed, *G*. *duodenalis* infection can be asymptomatic, or cause acute or chronic diarrhea, dehydration, intestinal malabsorption, malnutrition and steatorrhea [2, 4–7]. Chronic fatigue, post-infectious irritable bowel syndrome and intestinal dysbiosis have also been documented in humans as possible consequences of *G*. *duodenalis* infections, [28, 29], while growth retardation and cognitive malfunction have been reported in children from endemic areas [2, 30]. Probiotics may interfere with *G*. *duodenalis* infection through different mechanisms, including competition for limited adhesion sites, competition for nutrients that would otherwise be utilized by *G*. *duodenalis*, stimulation of the host immune response and by producing substances that may inhibit *G*. *duodenalis* [6, 7, 11]. Probiotic bacteria can produce compounds, which have inhibitory effects directed against pathogens, as viruses, bacteria, fungi, parasites, as well as against cancer cells [11]. Among them, the anti-*G*. *duodenalis* activity of probiotic compounds, mainly derived from *Lactobacilli*, has been demonstrated [11, 20, 24]. In fact, bacteriocins derived from *Lactobacillus acidophilus* were found able to inhibit *in vitro* the adhesion and the growth of *G*. *duodenalis* trophozoites [11]. Moreover, these negative effects were found associated with severe morphological changes of *G*. *duodenalis* trophozoites, a decline of the intestinal parasite density and amelioration of intestinal pathology in infected mice treated with *L*. *acidophilus* bacteriocins [11]. More recently, results from some studies suggested that the ability to deconjugate bile salts showed by some lactobacilli, as *L*. *johnsonii* strain LA1 and *Lactobacillus gasseri* CNCM I-4884, may represent a further mechanism contributing to the inhibition of *Giardia* trophozoite growth *in vitro* [31, 32].

Negative effects on *G*. *duodenalis* showed by the fresh supernatant of the commercial probiotic evaluated *in vitro* and *ex vivo* in the present study, are similar or higher to those reported in most of these previous studies. In fact, the fresh Slab51 supernatant was able to inhibit *in vitro* the adhesion and the growth of *G*. *duodenalis* trophozoites, although this inhibition was significantly lower than that of metronidazole. In the study of Perez *et al*. [24], the culture supernatant of the probiotic strain LA1 of *Lactobacillus johnsonii* was able to control *G*. *duodenalis* growth *in vitro* but it was unable to inhibit the adhesion of the parasite, while six *Lactobacillus acidophilus* strains tested did not show any noticeable effects. These data could be indicative that Slab51 constituent probiotic strains, mainly *L*. *plantarum* DSM 32244, possibly produce more effective active anti-*G*. *duodenalis* compounds with respect to those produced by *L*. *johnsonii* LA1. Negative effects on *G*. *duodenalis* adherence here observed are consistent with the ability reported for some lactobacilli to modulate *G*. *duodenalis* infection *in vivo* by minimizing or preventing the adherence of trophozoites to the intestinal mucosal surface [33, 34].

In agreement with previous studies in which some probiotic compounds were found able to induce morphological changes of *G*. *duodenalis* trophozoites [11, 24], important morphological alterations of this protozoan parasite were herein observed in *G*. *duodenalis* trophozoites from *in vitro* cultures treated with Slab51 fresh supernatant, including profound alterations of cellular and nuclear membranes, nuclear disorganization and formation of intra-cytoplasmic cavities. These *in vitro* cytopathic effects are very similar to those caused by *L*. *acidophilus* bacteriocins *in vivo*, possibly indicating a similar mode of action [11] and may be one of the main factors responsible for the inhibition of the trophozoite proliferation and adhesion here observed *in vitro*.

Trials performed in this study showed that the *in vitro* inhibiting effects on *G*. *duodenalis* showed by Slab51 fresh supernatant were greatly reduced by heat treatment at 56°C and completely annulled at 90°C, indicating that active metabolites contained in Slab51 supernatant are likely term labile compounds. These results agree with those of a previous report [24] and encourage further studies aimed to identify the extracellular factors responsible for the anti-*Giardia* effects of fresh Slab51 supernatant observed in the present study.

Results from *ex vivo* trials confirmed the inhibiting effects of the fresh supernatant of Slab51 on *G*. *duodenalis* trophozoites observed *in vitro*. The best observations were obtained after an 18h incubation period. In fact, after this period a significant reduction of *G*. *duodenalis* trophozoites and a significantly greater vitality of intestinal epithelial cells was evidenced in treated intestinal cultures respect to the untreated controls. Moreover, *G*. *duodenalis* was found capable of slowing or damaging the intestinal epithelial cell turnover in untreated *ex vivo* cultures, since in these cultures the apoptotic rate was increased. On the other side, obtained results showed that the number of *G*. *duodenalis* trophozoites was significantly lowered by the fresh Slab51 supernatant. Moreover, in *ex vivo* cultures the apoptosis and the death of intestinal epithelial cells was higher in *G*. *duodenalis-*inoculated cultures respect to those inoculated with *G*. *duodenalis* and the fresh supernatant of Slab51, indicating that the damage to the epithelial cells induced by *G*. *duodenalis* was reduced by the fresh supernatant of this commercial probiotic. Considering that the increase in the rate of enterocyte apoptosis and enterocyte damage are included among the main pathogenic mechanisms of *G*. *duodenalis* [34], results obtained in *ex vivo* trials are promising about possible *in vivo* protective effects of the fresh culture supernatant of Slab51 against *G*. *duodenalis*. This is the first study in which negative effects on *G*. *duodenalis* by metabolites from lactobacilli were demonstrated both *in vitro* and by using a murine *ex vivo* model. In previous studies, a variety of different systems have been used to evaluate the adherence and growth of *G*. *duodenalis*, including synthetic surfaces, human cells and non-human cells, as isolated rat enterocytes and rat enterocyte cell lines [35–37]. Among them, the human colonic adenocarcinoma derived epithelial cell line Caco-2, functionally and structurally may resemble small bowel enterocytes [38]. Therefore, this cell line model is considered useful and appropriate for studies of host intestine-pathogen interactions, and it is frequently used as a model to study the attachment and other effects of *G*. *duodenalis* trophozoites under different conditions [35]. However, this model does not allow a proper evaluation of the damages caused by *G*. *duodenalis* trophozoites to the intestinal mucosa and of associated inflammatory cells, while the murine *ex vivo* model performed in this study allowed these evaluations, by preserving the tissue architecture and the cellular complexity over several days. Indeed, alterations, damage and inflammation herein observed in *ex vivo* negative controls, i.e. intestinal tracts inoculated with *G*. *duodenalis* trophozoites only, were not so different from that observed in *in vivo* rodent models and at histopathological examination of intestinal biopsies taken from symptomatic human patients [30, 35]. Moreover, the *ex vivo* mouse model used in this study allowed to assess the anti-*Giardia* activity of the fresh supernatant of Slab51 by the evaluation of several positive effects on intestines inoculated with *G*. *duodenalis* trophozoites, although this model cannot be able to mimic the complexity of whole living organisms.

In conclusion, results from this study showed that the fresh culture supernatant of the commercial probiotic Slab51 has negative effects on *G*. *duodenalis* both *in vitro* and *ex vivo* in a mouse model. These antagonistic effects may suggest that this probiotic may likely represent a further and interesting approach for the prevention of giardiasis and/or the reduction of the pathogenic effects and proliferation of this protozoan parasite in infected hosts. However, further studies aimed to evaluate its efficacy *in vivo* on experimentally and/or naturally infected animals, are needed.
